# Ecosystem Metabolic Rates Estimated from Diel Oxygen Measurements in Two Subtropical Estuaries

**DOI:** 10.1007/s12237-025-01597-y

**Published:** 2025-08-07

**Authors:** J. M. Arriola, R. G. Najjar, H. Briceño, C. Hu, M. Herrmann, M. W. Beck

**Affiliations:** 1 The Pennsylvania State University, University Park, PA USA; 2 Florida International University, Miami, FL USA; 3 University of South Florida, St. Petersburg, FL USA; 4 Tampa Bay Estuary Program, St. Petersburg, FL USA

**Keywords:** Estuary Metabolism, Primary Production, Subtropical Estuaries, Dissolved Oxygen

## Abstract

Subtropical estuaries worldwide are facing increasing pressure from human population growth, development, and climate change. Carbon is a useful currency for understanding how estuaries respond to these pressures and yet relatively little is known about carbon cycling in subtropical estuaries. Here we compute gross primary production (GPP), ecosystem respiration (ER), and net ecosystem production (NEP) from the diurnal cycle in dissolved oxygen measured during 38 week-long individual deployments over three years in two estuaries in the southeastern United States, Biscayne Bay and Tampa Bay. On average for both estuaries, GPP and ER nearly balance, with NEP about an order of magnitude smaller. Even though production in Tampa Bay and Biscayne Bay is dominated by different primary producers and limiting nutrients, mean GPP was the same, about 190 mmol O_2_ m^–2^ d^–1^ (570 g C m^–2^ y^–1^). Our GPP estimates for Biscayne Bay are more than an order of magnitude greater than the only other productivity estimates available for this system, which are planktonic net primary productivity measurements from the late 1970s. GPP was strongly correlated with water temperature in Biscayne Bay (*r* = 0.60) but had the strongest correlation with salinity in Tampa Bay (*r* = 0.39). These findings highlight the importance of more frequent production measurements in these complex estuaries, especially in the face of a changing climate.

**Supplementary Information:**

The online version contains supplementary material available at 10.1007/s12237-025-01597-y.

## Introduction

**Fig. 1:**
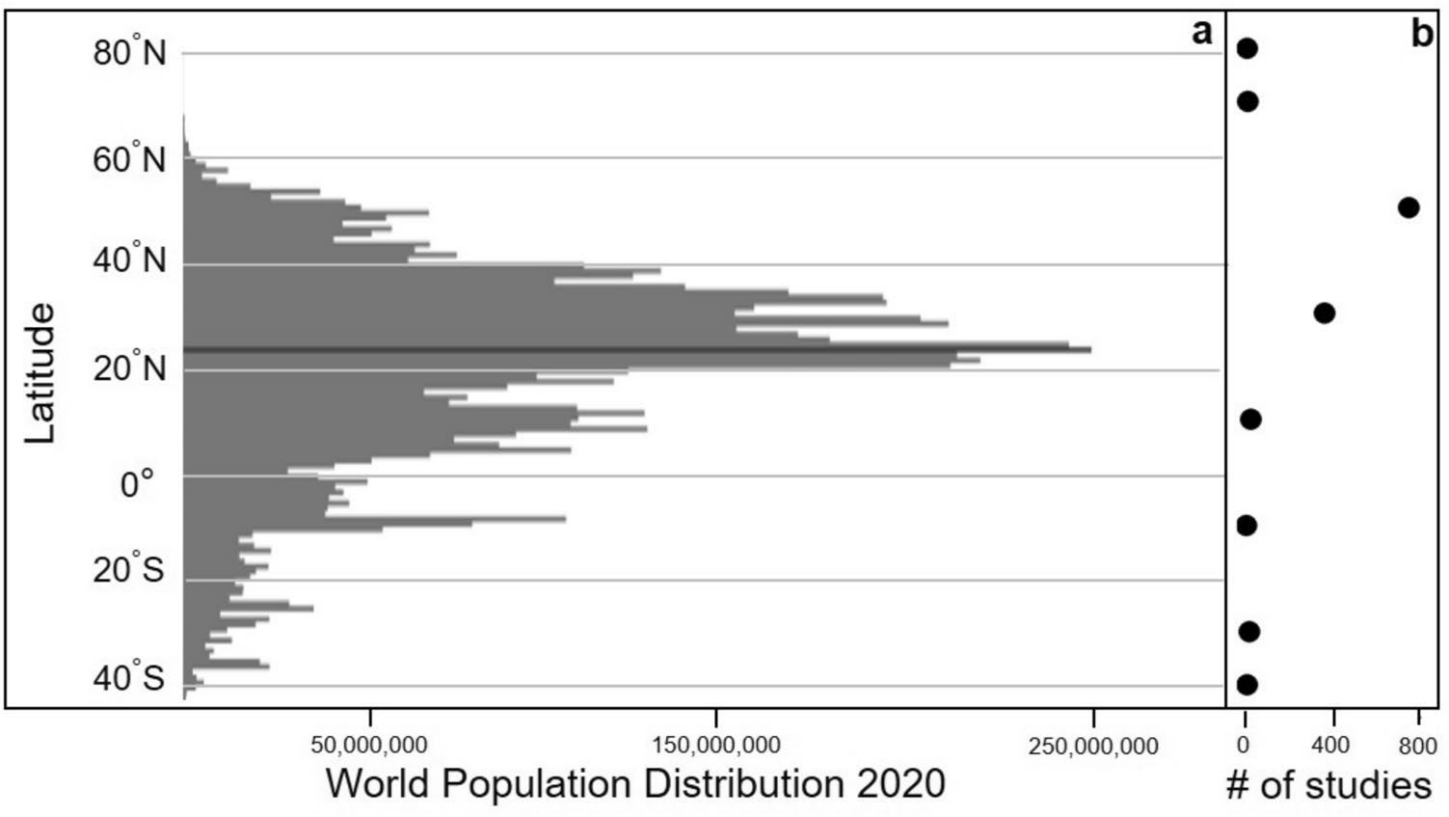
Distribution by latitude of **a** world population in 2020 per degree latitude (adapted from statsmapsnpix.com using data from worldpop.org, 2018) and **b** number of annual phytoplankton primary production studies in 20° latitude bands using data from Cloern et al. (2014)

Subtropical estuaries worldwide are facing increasing pressure from human population growth and urban development, such as nutrient enrichment (Pagliosa et al., 2006), and climate change, such as sea-level rise (Fox-Kemper et al., 2021). Figure 1a shows that the world’s population is concentrated in the subtropics of the Northern Hemisphere. The projected global population for the year 2050 is 9.7 billion people with most of the growth in India and Sub-Saharan Africa, both of which reside in the tropics and subtropics (United Nations 2019). On average, subtropical waters are becoming saltier, which is attributed to increases in evaporation minus precipitation and sea-level rise, and the trend is likely to continue due to further intensification of the hydrological cycle expected under increases in greenhouse gases (Costa et al., 2023; Fox-Kemper et al., 2021). The atmosphere’s Hadley circulation, which is the dominant overturning circulation in the tropics and subtropics, is extending poleward and intensifying (Davis & Rosenlof, 2012). This trend is projected to continue (Hu et al., 2013), which will likely have further impacts on the hydrological cycle in subtropical estuaries. Finally, subtropical coastal regions are where tropical cyclones make landfall, causing the most damage. A recent Intergovernmental Panel on Climate Change report suggests the frequency of intense tropical storms will increase substantially in some ocean basins and shift poleward as greenhouse gases continue to rise (Seneviratne et al., 2021). Given that estuaries have important economic, ecological, and biogeochemical functions (e.g., fisheries, recreation, habitat, and water quality improvement), it is critical to develop a quantitative understanding of how estuaries respond to external perturbations. Such an understanding is particularly critical for subtropical estuaries, which serve much of the human population and face distinct stresses from development and climate change.

As noted by Kemp and Testa (2011), “the metabolism of a coastal ecosystem represents an integrated measure of the vitality of that system in terms of overall rates of production and consumption of organic matter.” Ecosystem metabolism is quantified by gross primary production (GPP), ecosystem respiration (ER), and their difference, net ecosystem production (NEP = GPP – ER), which is also often referred to as net ecosystem metabolism (Odum, 1956). NEP is positive for net autotrophic conditions and negative for net heterotrophic systems. Knowledge of a system’s NEP is often the key factor in determining whether a region is a net source or sink of a wide variety of key constituents, including organic carbon, carbon dioxide, oxygen, and inorganic nutrients. Long-term, high-resolution time series of estuarine water quality data, such as those available from the United States National Estuarine Research Reserves (NERRs), are often used to quantify ecosystem metabolism (e.g., Baumann & Smith, 2018; Caffrey et al., 2014). However, the fixed monitoring stations within these estuarine reserves are typically situated in nearshore, shallow waters (Baumann & Smith, 2018), and therefore are likely missing the full spatial integration of metabolism in these coastal systems (Staehr et al., 2012; Caffrey et al., 2014).

A global synthesis of annual phytoplankton primary production (APPP) estimates in estuaries by Cloern et al. (2014) shows that there are fewer metabolism measurements made in the more populous tropics and subtropics compared with the less populous northern mid-latitude regions, highlighting the need for more metabolic studies in tropical and subtropical estuaries (Fig. 1b). Specifically, in the Northern Hemisphere in the year 2020, the tropics and subtropics (0–40°N) encompassed about 73% of the human population and only about 25% of estuarine APPP estimates (www.worldpop.org, accessed Apr 25 2022). In contrast, the region between 40 and 60°N contained only 15% of the human population but about 60% of estuarine APPP estimates. In addition, most of the studies in the global synthesis were conducted in the 1990 s, and therefore are likely not representative of modern estuaries and their current ecological state, which may have changed over the past thirty years due to such factors as restoration efforts, increased urban pressures, and climate change.

More recently, estuarine metabolism studies have been conducted in subtropical zones in South Africa (Tagliarolo & Scharler, 2018), the Americas (Bordin et al., 2024a, 2024b; Brandini et al., 2019; Cabral & Fonseca, 2019; Medina-Galván et al., 2021; Murrell et al., 2018; Shipley et al., 2022; Soria-Píriz et al., 2017), and China (Shen et al., 2022). Although these more recent studies use a variety of techniques ranging from the diurnal oxygen variation method (Odum, 1956; referred to as “the Odum method” hereafter) (Soria-Píriz et al., 2017; Murrell et al., 2018; Tagliarolo & Scharler, 2018; Brandini et al., 2019; Shen et al., 2022; Shipley et al., 2022; Bordin et al., 2024a) to the Land–Ocean Interactions in the Coastal Zone (LOICZ) mass-balance model (Bordin et al., 2024b; Cabral & Fonseca, 2019; Medina-Galván et al., 2021; Swaney et al., 2011), all recognize the importance of understanding relationships between increasing anthropogenic pressures and the trophic status of coastal and estuarine waters. While these studies have added to our knowledge of subtropical estuarine metabolic rates and their driving factors, they are relatively few in number. Therefore, there is a need for similar studies in other regions. The goal of the present study is to provide the first system-wide estimates of GPP, ER, and NEP of two subtropical estuaries in the southeastern United States, Tampa Bay and Biscayne Bay. Specific research questions we seek to answer are: (1) To what extent do GPP and ER balance? (2) Do the different primary producers and limiting nutrients in the two systems lead to different metabolic rates? (3) What are the environmental factors that are most highly correlated with metabolic rates? To answer these questions, we conducted numerous field deployments throughout the open waters of the two estuaries, focusing on the diurnal cycle of dissolved oxygen, and employed the Odum method to estimate metabolic rates.

## Methods

### Study Area

#### Biscayne Bay

**Fig. 2:**
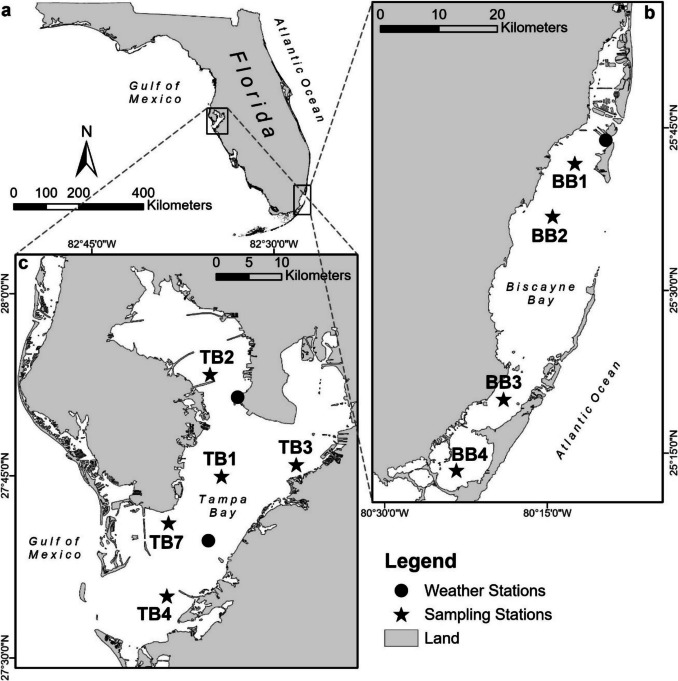
**a** Locations of Biscayne Bay and Tampa Bay. Locations of water sampling stations and weather stations in **b** Biscayne Bay and **c** Tampa Bay

Biscayne Bay is a subtropical, oligotrophic, shallow estuary located in southern Florida (Fig. 2). Biscayne Bay has a length of about 55 km, extends approximately 12 km from shore at its widest point, and encompasses a total area of approximately 700 km^2^ (Caccia & Boyer, 2005). Biscayne Bay receives freshwater through discharge from the Miami River with a mean discharge of about 5 m^3^ s^−1^ (waterdata.usgs.gov, accessed Jun 28 2025), numerous canals managed by the South Florida Water Management District, precipitation, and submarine groundwater discharge (Stalker et al., 2009; Miami-Dade County, 2019). Because its mean depth is only 2.5 m, the bay tends to be well-mixed; the water residence time based on the salt balance is about 14 days (Seidensticker et al., 2019).

In recognition of its valuable natural attributes, almost all portions of Biscayne Bay have been classified as a National Park, Outstanding Florida Waters, a State Aquatic Preserve, and a State critical wildlife area (Miami-Dade County, 2019). Biscayne Bay is a productive estuary and supports many shallow, saltwater fisheries, such as pink shrimp and bonefish (Rezek et al., 2023). However, Biscayne Bay’s proximity to Miami-Dade county (population ~ 2.7 million) and the Homestead Air Force Base and Agricultural Area to the south has caused widespread environmental changes since the beginning of the twentieth century. Anthropogenic influences on the bay have caused or contributed to increased nutrient loading and algal blooms, septic systems submerged by sea level rise, sediment contamination, fuel seepage from vessels, habitat destruction, and species loss (Caccia & Boyer, 2005; Miami-Dade County 2018; Miami-Dade County, 2019; Millette et al., 2019). Biscayne Bay is generally oligotrophic as indicated by low phosphorus and chlorophyll a concentrations (Brand et al., 1991; Millette et al., 2019).

Biscayne Bay has historically been partitioned into three regions: North, Central, and South, with the following characteristics (Caccia & Boyer, 2005). In the North Bay, the effects of external nutrient loading from urban development are amplified by poor circulation with the ocean from land barrier structures to the east and the coastline to the west (Millette et al., 2019). Phytoplankton abundance is much higher with more frequent, large blooms in the North Bay than in the other regions (Brand et al., 1991). The Central Bay exchanges more water with the Atlantic Ocean because, unlike the North and South bays, it is not enclosed by barrier islands or other structures (Wang et al., 2003). Thus, water residence time is lower, explaining, in part, why the water quality of the Central Bay is higher than in the adjacent regions. However, the Central Bay is still influenced by urbanization because the Miami River, which drains a watershed encompassing suburbs of Miami, empties into the northern portion of the region. While the South Bay is the least developed of the three regions and harbors more healthy marine communities, it is also impacted by anthropogenic factors, such as agricultural nutrient runoff and contaminated sediments originating from Military Canal and other canals (Caccia & Boyer, 2005). More recent finer subdivisions within the three primary regions of Biscayne Bay highlight significant gradients in nutrient concentrations and turbidity from the west side of the bay to the east (Briceño et al., 2013). Seagrass distribution also varies across the regions, with extensive seagrass beds particularly in the Central and South Bays (Miami-Dade County, 2019).

We are unaware of previous GPP estimates in Biscayne Bay, although published net primary production (NPP = GPP minus autotrophic respiration) and NEP estimates are available. In 1977, Roman et al. (1983) observed planktonic NPP to vary laterally across the middle of Biscayne Bay from 13 g C m^–2^ y^–1^ near the open ocean to 46 g C m^–2^ y^–1^ near shore. Roman et al. (1983) estimated that over 90% of the primary production in Biscayne Bay was from seagrass and attached algae based on a study by Odum (1957). Odum (1957) found that the dominant seagrass species in the sea-exposed Florida Keys, *Thalassia testudinum* (also dominantly found in Biscayne Bay), produced between 520 and 640 g C m^–2^ y^–1^, an order of magnitude higher than planktonic NPP measured by Roman et al. (1983). Another study based on historical monthly oxygen surveys found that NEP in Biscayne Bay had modest seasonality, with the lowest NEP typically occurring in September, and the long-term average NEP over 12 years was –5.3 mmol O_2_ m^–2^ y^–1^, or –44 g C m^–2^ y^–1^ assuming C:O_2_ of 106:154 (Hedges et al., 2002), indicating net heterotrophy (Seidensticker et al., 2019).

#### Tampa Bay

Tampa Bay is located along the west coast of central Florida with a population of just over 3 million people living within its watershed (Tampa Bay Estuary Program, 2022) (Fig. 2). It has a surface area of ~ 1000 km^2^, which is slightly greater than that of Biscayne Bay, an average water depth of ~ 4 m, a water residence time of about 100 days, and is considered to be partially to well-mixed with a residual circulation consisting of surface seaward flow and deep landward flow in its dredged channels, which are typically ~ 15 m in depth (Weisberg & Zheng, 2006). The major tributaries to Tampa Bay are the Hillsborough, Alafia, Little Manatee, and Manatee Rivers with mean discharge rates ranging between 5 to 11 m^3^ s^−1^ (waterdata.usgs.gov, accessed Jun 21 2025). The estuary provides vital habitat for fisheries-relevant species, such as crustaceans, Flounder, Red Drum, oysters, and a variety of marine mammals (Tampa Bay Estuary Program, 2022). As of 2023, about 1 in 10 jobs in the Tampa Bay watershed are supported by bay-dependent industries (Todd et al., 2023). With three seaports, Tampa Bay is considered to be one of the busiest coastal port regions in terms of cargo tonnage in the US (United States Army Corps of Engineers, 2018).

Tampa Bay is considered to be a success story for estuarine restoration facilitated by the USEPA-administered National Estuary Program and its regional partners. Residential and industrial development in the bay’s watershed from the 1950 s to the 1980 s resulted in excess nutrient and sediment inputs (e.g., untreated or poorly treated sewage), which led to dramatic water quality declines, including high chlorophyll levels, high turbidity, and a decline in seagrass coverage to almost half the pre-development average (Sherwood et al., 2016). Poor water quality prompted the Environmental Protection Commission of Hillsborough County to initiate bay-wide water quality monitoring in 1972. Currently, surface waters flowing into the bay are subject to state and federally recognized thresholds for nutrients known as total maximum daily loads (floridadep.gov/TMDL, Accessed May 10 2024). The estuary is primarily nitrogen-limited due to the underlying geology of the region, which is rich in phosphates (Greening et al., 2014).

Efforts to reduce the external nitrogen load have been highly successful, resulting in greater water clarity during the past two decades, with surface chlorophyll reduced by half compared with the 1980–1985 levels (Johansson, 2010; Johansson & Greening, 1999; Tomasko et al., 2005). Greater water clarity resulted in increases in seagrass coverage throughout the bay. In 2014, seagrass coverage reached the recovery goal of 160 km^2^, set in 1995 based on the 1950 distribution (Sherwood et al., 2016), and peaked at an all-time high in 2016 at 169 km^2^. However, seagrass coverage declined from 2016 to 2022 by 28%, primarily in Old Tampa Bay. There is no consensus yet on the cause of this decrease (tampabay.wateratlas.usf.edu/seagrass-monitoring, accessed Feb 14 2024), although temperature and salinity changes related to climate change have been implicated (Beck et al., 2024).

Similar to Biscayne Bay, we are unaware of any historical GPP estimates for Tampa Bay, though there are measurements of NPP. Johansson (2010) made monthly planktonic NPP measurements at four locations in Tampa Bay, with production highest in the summer months and annual production declining from around 700 g C m^–2^ yr^−1^ in the 1980 s to around 400 g C m^–2^ yr^−1^ in the 2000s. Xu et al. (2022) used MODIS satellite data and a machine learning algorithm trained on data from Johansson (2010) to estimate NPP for all of Tampa Bay from 2002 to 2020. These authors found NPP to increase by 14% over the study period, largely as a result of warming.

### Study Approach

Metabolic rates were computed using the R package *WtRegDO* (Beck et al., 2015; github.com/fawda123/WtRegDO; Murrell et al., 2018), which is based on the oxygen mass balance method of Odum (1956). *WtRegDO* requires inputs of dissolved oxygen concentration, water temperature, salinity, water depth, barometric pressure, and wind speed. We made field measurements of the first four inputs and used available meteorological data for the barometric pressure and wind speed. To aid in the interpretation of the metabolic rate estimates, we characterized the water chemistry by measuring total nitrogen (TN), total phosphorus (TP), total organic carbon (TOC), and chlorophyll *a* (chl-*a*) from discrete water samples collected at the beginning and end of each deployment except during the Spring of 2021 in Biscayne Bay; all were measured in surface and bottom waters, except for chl-*a*, which was measured only in surface waters.

#### Continuous Oxygen Measurements

Deployments of probes that continuously measure dissolved oxygen in open waters are time-consuming and expensive, so we had to choose deployment locations and times carefully to maximize our chances at capturing key aspects of spatial and temporal variability. Typically, five deployments at four stations were performed in each estuary starting in the fall of 2017 and ending in summer 2021, resulting in a total of 38 individual deployments (Table 1 and Fig. 2). Criteria for station locations were to encompass the sub-regions and interior bays within both estuaries, shallower than 3 m, at least 3 km offshore, close to existing water quality monitoring stations if possible, and seagrass present if possible. The main criteria for deployment times was to capture wet and dry seasons as well as some interannual variation. For the study region, the wet season runs from May to October and the dry season from November to April (Duever et al., 1994). Our deployments reasonably satisfy our temporal variability criteria, with some deployments in the heart of the wet season and others at the shoulders of the dry season. Due to erosion of the seagrass beds at station TB4 in Tampa Bay (Lower Tampa Bay) during 2018, station TB7 (Lower Middle Tampa Bay) was used for deployments 3–5. Water column depths ranged between 2.4 and 4.4 m (mean of 3.5 m) in Biscayne Bay and 1.2 and 4.4 m (mean of 2.8 m) in Tampa Bay (Supplemental Table 1). Lengths of deployments ranged from 7 to 17 days (mean 11 days) in Biscayne Bay and from 2 to 15 days (mean 8 days) in Tampa Bay (Table 1). Notably, the final deployment period in Tampa Bay (April 26th to May 6th, 2021) occurred approximately two weeks after the emergency release of wastewater from Piney Point, a legacy mining facility near the southeast shore of Lower Tampa Bay. This release delivered 205 tons of nitrogen to Lower Tampa Bay over ten days, exceeding the typical annual external nitrogen load to that area of the bay (Beck et al., 2022).

**Table 1:** Deployment stations, locations, and periods in Biscayne Bay and Tampa Bay. N/A = Data not available

Estuary	Station ID	Station Name	Latitude (°)	Longitude (°)	Deployment	Date Ranges
Biscayne Bay	BB1	Key Biscayne	25.695389	−80.207667	1	11/06/2017—11/15/2017
					2	6/20/2018—6/28/2018
					3	4/1/2019—4/16/2019
					4	10/29/2019—11/08/2019
					5	6/02/2021—6/11/2021
	BB2	North Biscayne National Park	25.614028	−80.241806	1	11/06/2017—11/15/2017
					2	6/20/2018—6/28/2018
					3	4/1/2019—4/16/2019
					4	10/29/2019—11/08/2019
					5	6/02/2021—6/11/2021
	BB3	Card Sound	25.332833	−80.317028	1	11/07/2017—11/15/2017
					2	6/21/2018—6/28/2018
					3	4/1/2019—4/12/2019
					4	10/29/2019—11/14/2019
					5	N/A
	BB4	Barnes Sound	25.223611	−80.389611	1	11/07/2017—11/15/2017
					2	6/20/2018—6/28/2018
					3	4/1/2019—4/16/2019
					4	10/29/2019—11/14/2019
					5	6/01/2021—6/11/2021
Tampa Bay	TB1	Middle Tampa Bay	27.749361	−82.571972	1	10/10/2017—10/17/2017
					2	7/18/2018—7/25/2018
					3	6/11/2019—6/13/2019
					4	11/20/2019—11/21/2019
					5	4/26/2021—5/06/2021
	TB2	Old Tampa Bay	27.889306	−82.587639	1	10/11/2017—10/18/2017
					2	7/17/2018—7/24/2018
					3	6/11/2019—6/14/2019
					4	11/21/2019—12/05/2019
					5	4/26/2021—5/06/2021
	TB3	Hillsborough Bay	27.765306	−82.469306	1	10/11/2017—10/13/2017
					2	7/17/2018—7/24/2018
					3	N/A
					4	11/20/2019—12/05/2019
					5	4/26/2021—5/06/2021
	TB4	Lower Tampa Bay	27.585222	−82.646917	1	10/12/2017—10/17/2017
					2	7/18/2018—7/25/2018
	TB7	Lower Middle Tampa Bay	27.685361	−82.644667	3	6/11/2019—6/16/2019
					4	11/20/2019—12/04/2019
					5	4/26/2021—5/06/2021

Sondes (YSI EXO2® for deplolyments 1–4 and HOBO® for deployment 5) were deployed 30 cm from the bottom to measure temperature, conductivity, water pressure, and dissolved oxygen at 10 min intervals. Conductivity measured by the HOBO® was converted to practical salinity units using the algorithm of Fofonoff & Millard (1983), which is the same one employed internally by the EXO2®. Measured water pressure by the HOBO® was converted to depth based on a constant atmospheric pressure of 1010 hPa, whereas the EXO2® internally converts water pressure to depth based on the atmospheric pressure at the time of calibration. Conductivity data were not collected at station BB3 during the summer 2021 deployment and no data were collected at station TB3 during the spring 2019 deployment; therefore production rate estimates are not available for these stations and times. The YSI® and HOBO® sensors were also used to collect vertical profiles of dissolved oxygen at the beginning of each deployment in order to assess the degree of stratification. All continuous and discrete sample data are available in an open access repository (Briceño et al., 2022).

#### Meteorological Data

Wind speed and barometric pressure data were downloaded from the National Oceanic and Atmospheric Administration (NOAA) National Data Buoy Center website (ndbc.noaa.gov, accessed Feb 18 2022). Data were collected every 6 min in Biscayne Bay at Virginia Key (station #8723214) and in Tampa Bay at Middle Tampa Bay and Old Port Tampa (stations #8726412 and #8726607, respectively) (Fig. 2). Meteorological data from the Old Port Tampa buoy were paired with water quality data recorded at station TB2 in Tampa Bay for all deployments due its close proximity to the buoy.

#### Metabolic Rate Estimates

We calculated vertically integrated GPP, ER, and NEP per metabolic day, the period between sunrises on two adjacent calendar days, using the mass balance equation for oxygen (Odum, 1956):

1 $$H\frac{d\left[{\text{O}}_{2}\right]}{dt}=GPP-ER+F$$

where *H* is the water depth, $\left[{\text{O}}_{2}\right]$ is the dissolved oxygen concentration, *t* is time, and *F* is the net flux of oxygen from the atmosphere. The water column is assumed to be well–mixed, an assumption supported by pre-deployment profiles of [O_2_]; the mean standard deviation of all pre-deployment profiles was 4 mmol O_2_ m^–3^, which is an order of magnitude less than the mean diel range of 45 mmol O_2_ m^–3^ observed during the deployments. A standard gas transfer model was used:

2 $$F={k}_{w}\left({\left[{\text{O}}_{2}\right]}_{sat}-\left[{\text{O}}_{2}\right]\right)$$

where ${k}_{w}$ is the gas transfer velocity for oxygen (computed from temperature, salinity, and wind speed; Wanninkhof, 2014) and ${\left[{\text{O}}_{2}\right]}_{sat}$ is the oxygen saturation concentration (computed within *WtRegDO* from barometric pressure, temperature, and salinity using Benson & Krause, 1984). ER is calculated from the nighttime oxygen budget assuming GPP is zero. GPP is computed from the daytime oxygen budget assuming the following night’s average value for ER. Daily mean NEP is the difference between mean daytime GPP and mean night-time ER (Beck et al., 2015). Thirty-minute averages of all parameters were computed before the model was applied, which reduced the sensitivity of the metabolic estimates to short-term fluctuations around sunrise and sunset. Ultimately, there were 19 occurrences of anomalous (i.e., negative) mean daily GPP or ER (9 in Biscayne Bay and 10 in Tampa Bay) which is 6% of the total number of individual GPP and ER estimates. All anomalous results are included in the station, deployment, and bay-wide averaged estimates. Depth measurements for all deployments were corrected by 30 cm to account for the height of the sensors off the bottom. Conversions from O_2_ to C used a C:O_2_ ratio of 106:154 (Hedges et al., 2002).

Lateral advection is not included in the mass balance (Eq. 1), which introduces errors into the metabolic calculations. Although the R package *WtRegDO* includes a method to remove the tidal component of the lateral advection signal in an estuarine dissolved oxygen time series, the method may not be suitable for deployments as short as a few days. After evaluation of salinity ranges, means, and standard errors for all stations and deployments, we assumed that transport errors were random and averaged out to zero over each deployment (Supplemental Table 2). Uncertainties are thus computed for the average metabolic rates during each deployment at each site and for all deployments and sites using the standard error of the mean (the standard deviation divided by the square root of the number of deployment days).

**Table 2:** Deployment averages of total nitrogen (TN), total phosphorus (TP), total organic carbon (TOC), and Chlorophyll *a* (Chl-*a*). N/A indicates data not available

	TN (mmol m^−3^)	TP (mmol m^−3^)	TOC (mmol m^−3^)	Chl-*a* (mg m^−3^)
Biscayne Bay				
Fall 2017	29.6	0.35	325	1.13
Summer 2018	26.7	0.27	334	0.82
Spring 2019	20.6	0.25	215	0.31
Fall 2019	23.5	0.21	272	0.56
All Deployments	25.1	0.27	288	0.70
Tampa Bay				
Fall 2017	24.9	5.7	429	10.26
Summer 2018	26.4	6.3	384	8.06
Spring 2019	23.6	3.8	306	2.55
Fall 2019	19.9	2.9	318	3.13
Spring 2021	21.2	2.9	N/A	3.11
All Deployments	23.2	4.3	348	5.20

## Results

### Continuous Oxygen Measurements

**Fig. 3:**
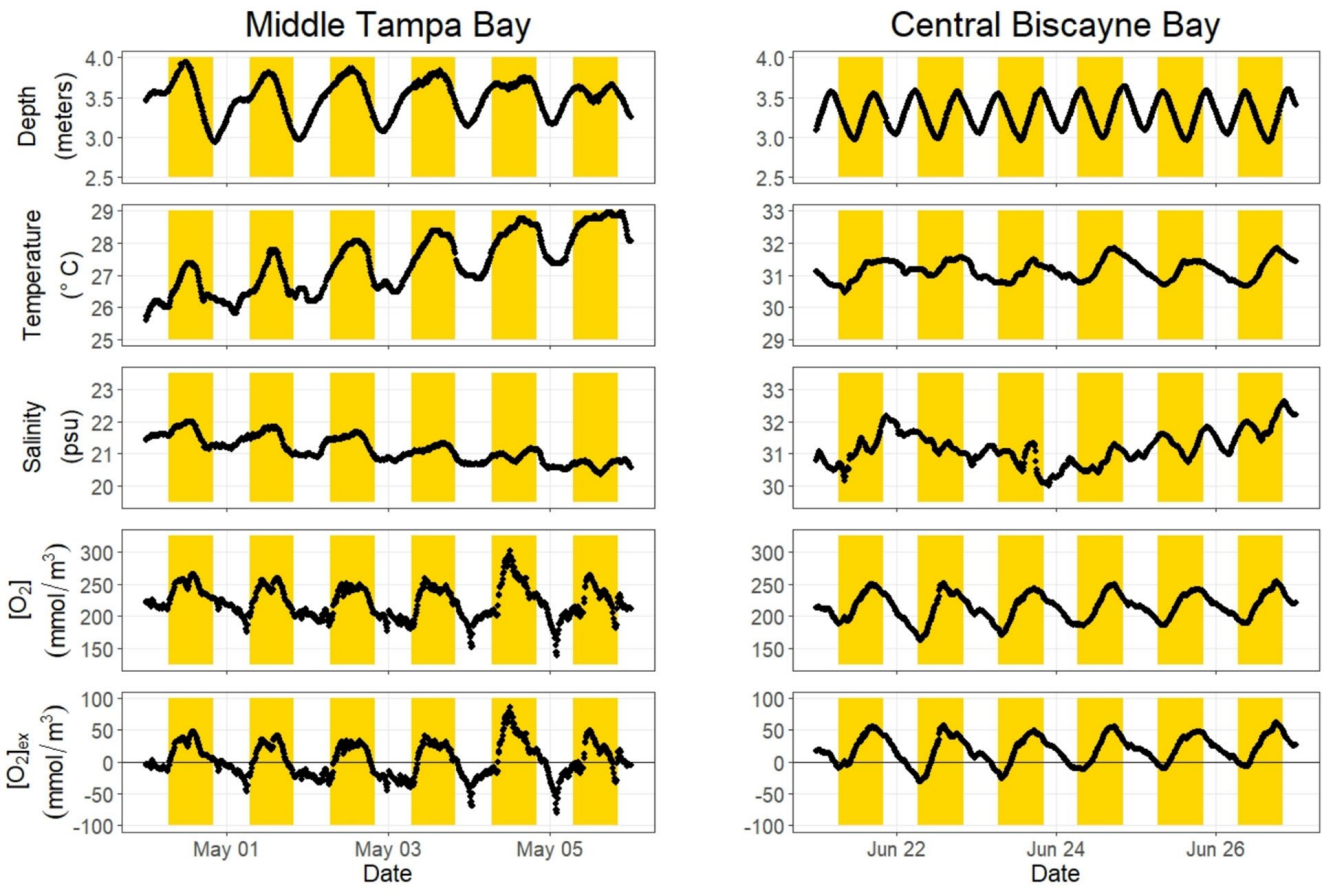
Example time series of depth, temperature, salinity, dissolved oxygen, and dissolved oxygen excess (above saturation) at 10-min intervals in Middle Tampa Bay (Station TB1, May 2021, left panels) and central Biscayne Bay (Station BB2, June 2018, right panels). Daytime hours are highlighted in yellow

Minimum, maximum, and means (± 2 standard errors) for water temperature, salinity, and [O_2_] measurements for both bays are provided in Supplemental Table S2. Representative time series are shown in Fig. 3 for temperature, salinity, depth, [O_2_], and O_2_ excess over saturation ([O_2_]_ex_) for both estuaries. Temperature and oxygen vary diurnally, reflecting the influence of radiative forcing (warming and an increase in oxygen due to net photosynthesis during the day, and cooling and a decrease in oxygen due to net respiration at night). Depth and salinity show clear influence of diurnal tides in Tampa Bay and semi-diurnal tides in Biscayne Bay.

### Water Chemistry

Means for TN, TP, TOC, and chl-*a* concentrations for all stations per deployment are provided in Table 2. TN, TP, and TOC for all stations in Biscayne Bay had means (± 2 standard errors) of 25.1 ± 2.5, 0.27 ± 0.02, and 288 ± 31 mmol m^−3^, respectively. TN and TOC in Biscayne Bay tended to be highest at the southernmost station and decreased northward. TN, TP, and TOC concentrations decreased from 2017 to 2019. The deployment-averaged TN:TP was consistently well above the canonical value of 16 (Redfield et al., 1963). Chl-*a* ranged between 0.15 and 1.6 mg m^–3^ with a mean of 0.70 ± 0.1 mg m^–3^. Chl-*a* concentrations were higher in the fall 2017 deployment and lowest in spring 2019.

In Tampa Bay, mean TN was 23.2 ± 1.6 mmol m^−3^ and TP had a mean of 4.3 ± 0.5 mmol m^−3^. Both TN and TP tended to be highest for all deployments at stations TB2 and TB3. TOC varied little across stations and depths with a mean of 348 ± 20 mmol m^−3^, though TOC was not measured for Spring 2021. The deployment-averaged TN:TP was consistently well below the canonical value of 16 (Redfield et al., 1963). Chl-*a* ranged between 0.62 and 14.05 mg m^–3^ with a mean of 5.20 ± 0.9 mg m^–3^. Chl-*a* and TP were consistently an order of magnitude lower in Biscayne Bay than Tampa Bay.

### Metabolic Rate Estimates

**Fig. 4:**
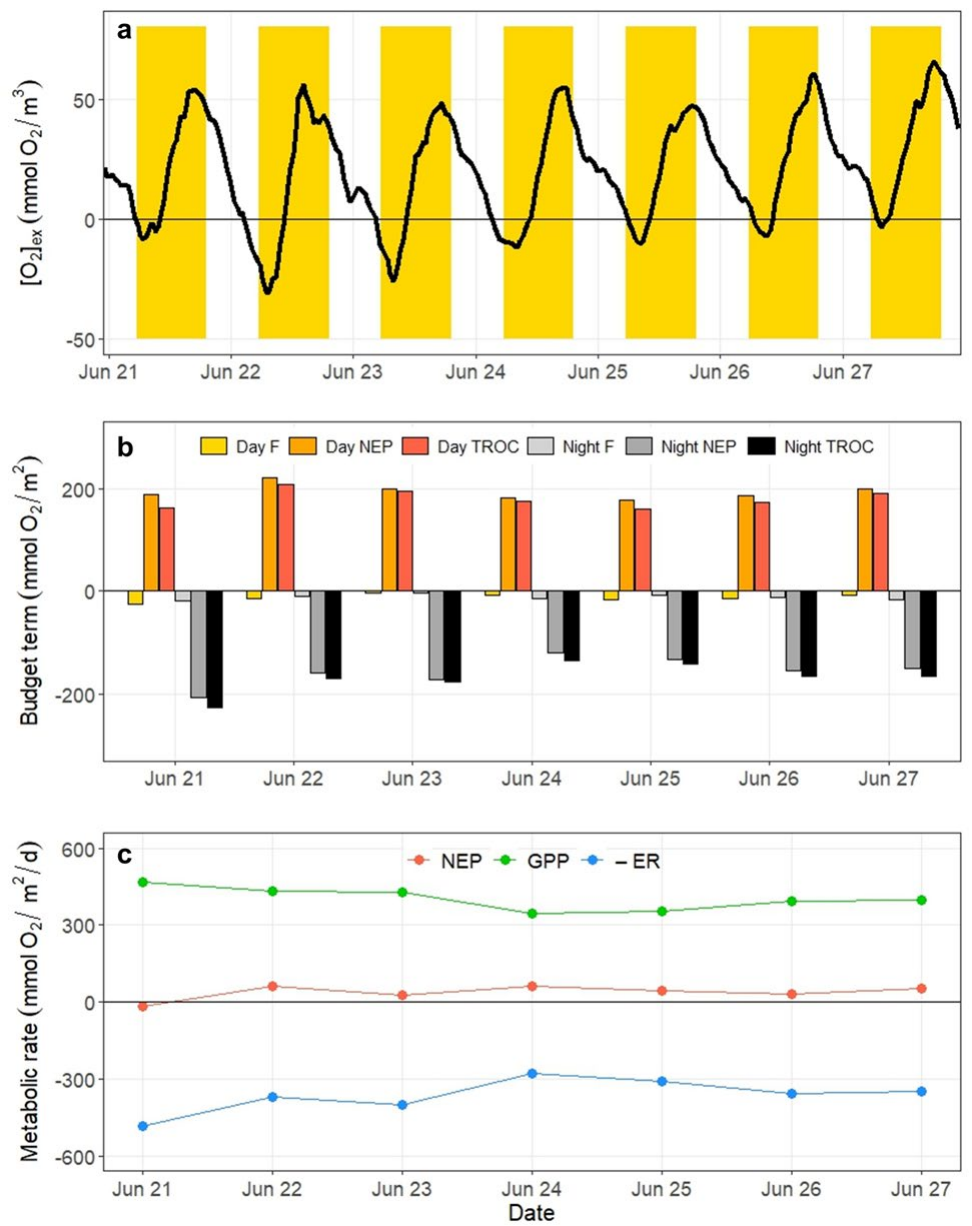
Example of rate calculations for central Biscayne Bay, Station BB2, June 2018. **a** Dissolved oxygen excess with daylight hours highlighted in yellow. **b** Integrals of air–sea flux (F), net ecosystem production (NEP), and the time rate of change of oxygen (TROC) for daytime and nighttime hours for each metabolic day. **c** Daily averages of gross primary production (GPP), ecosystem respiration (–ER), and NEP

An example of typical mass balance model results, using central Biscayne Bay in summer 2018, is shown in Fig. 4. Oxygen excess shows clear diurnal variability over the one-week deployment period (Fig. 4a). The dominant non-biological budget term is the rate of oxygen change between day and night, whereas gas transfer is relatively small (Fig. 4b). The daily averaged GPP and ER nearly balance, as illustrated by the low NEP (Fig. 4c).

**Fig. 5:**
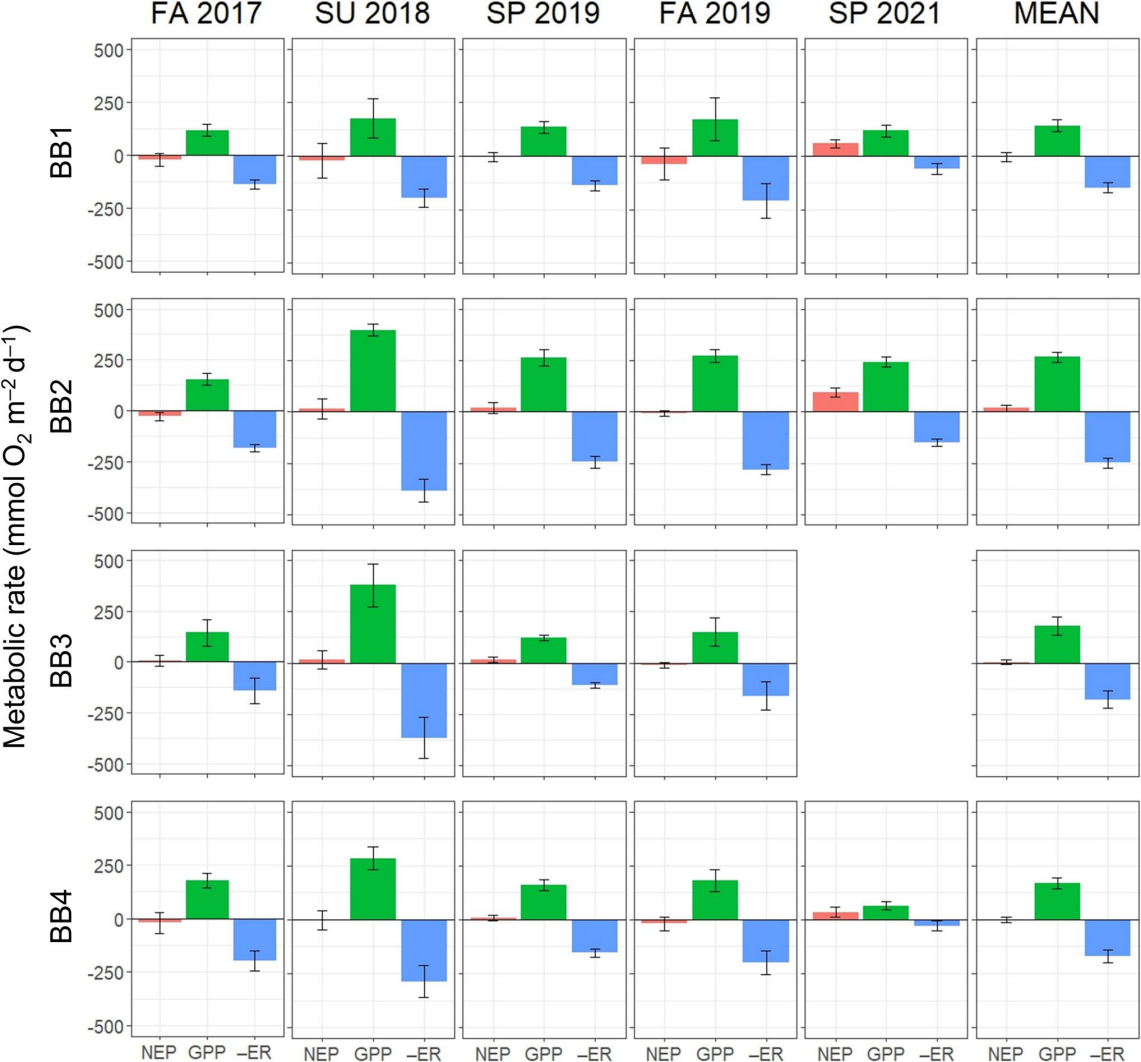
Average gross primary production (GPP), ecosystem respiration (–ER), and net ecosystem production (NEP) daily mean rates ± 2SE at each of the four stations in Biscayne Bay for each of the five deployments

**Fig. 6:**
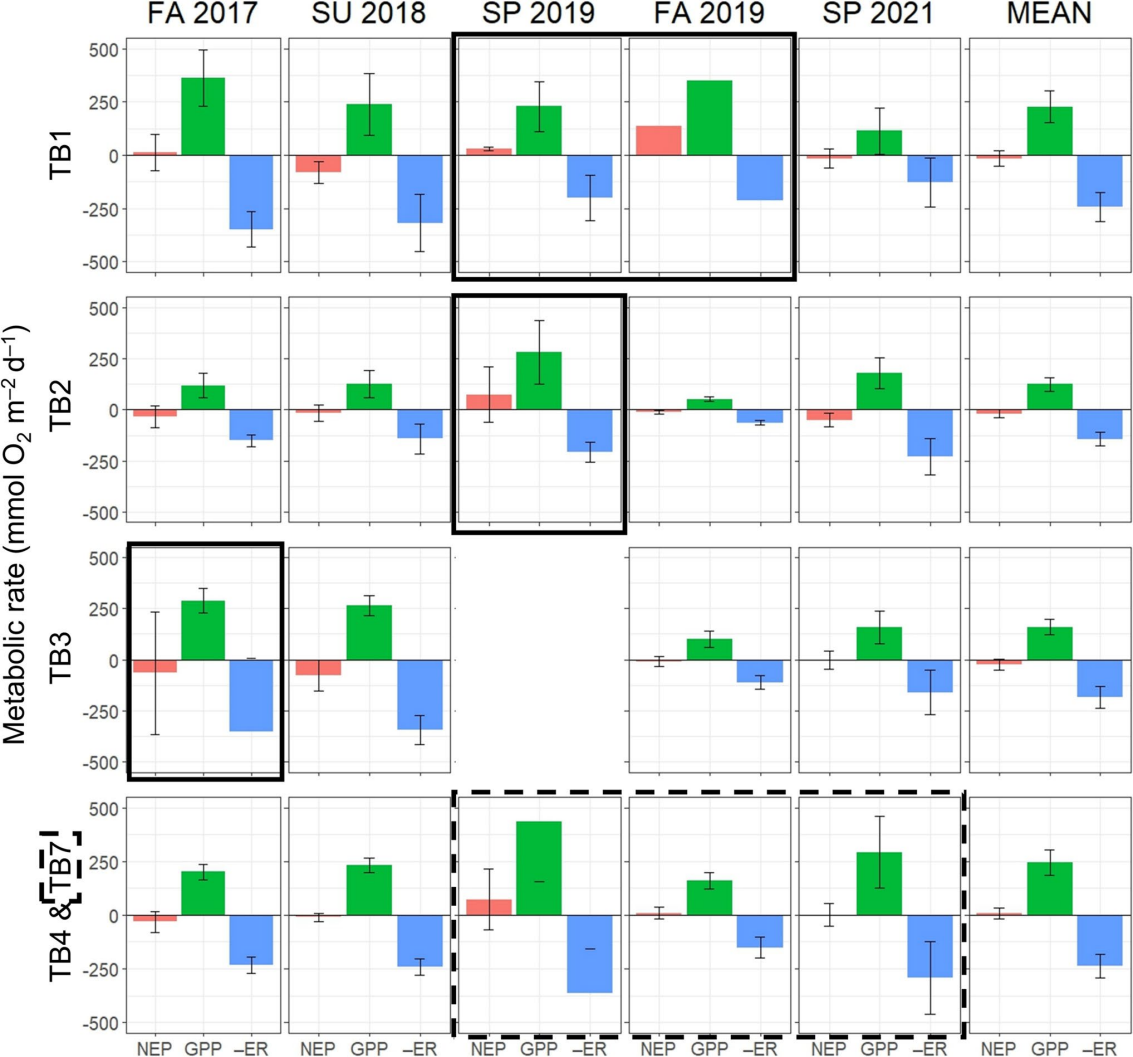
Average gross primary production (GPP), ecosystem respiration (–ER), and net ecosystem production (NEP) daily mean rates ± 2SE at each of the four sites in Tampa Bay for each of the five deployments and the mean for all deployments per site; stations TB4 and TB7 are averaged together. Graphs outlined in solid black boxes are averages where n < = 4 days. Graphs outlined in the dashed box are from station TB7

Mean daily rates of GPP, ER, and NEP averaged for each site per deployment period and the mean for all deployments per site for Biscayne Bay and Tampa Bay are shown in Figs. 5 and 6, respectively. These averages also show that GPP and ER nearly balance each other, resulting in NEP of relatively low magnitude. Deployments within both bays tended to be metabolically neutral, with an average negative NEP in about 50% and 60% of the sampling deployments for Biscayne Bay and Tampa Bay, respectively. Using the non-parametric Kruskal–Wallis test (Kruskal & Wallis, 1952), mean GPP and ER for all deployments are statistically different at station BB2 in central Biscayne Bay from the other stations (p-value < 0.001 for both GPP and ER). For stations in Tampa Bay, the only significant differences in mean GPP and ER are between station TB2 and the lower and middle sections of Tampa Bay, stations TB1, TB4, and TB7 (p-values < 0.05). Bay-wide deployment average metabolic rates are shown in Table 3. In Biscayne Bay, average GPP during summer 2018 was roughly twice that of the other four deployments. By contrast, average GPP in Tampa Bay during spring 2019 was more than 3 times that of the least productive deployment, fall 2019. NEP is net heterotrophic during both fall deployments in Biscayne Bay, but deployments in Tampa Bay are all net heterotrophic except for late spring 2019. However, the spring and fall 2019 NEP results for Tampa Bay may not be representative due to fewer sampling days across stations, such as only one full metabolic day being monitored in fall 2019 at station TB1, and missing data from station TB3 (Hillsborough Bay) in spring 2019 as TB3 was net heterotrophic during all other deployments (Fig. 6).

**Table 3:** Average metabolic rates (± 2SE) in each estuary for each of the five deployments and average rates for all deployments. Carbon rates were calculated using a C:O_2_ ratio of 106:154 ( Hedges et al., 2002). Includes results from Johansson (2010), Roman et al. (1983), and Seidensticker et al. (2019)

Estuary	Deploy #	Season	GPP	ER		NEP	GPP	ER	NEP
			mmol O_2_/m^2^/day (± 2SE)				g C/m^2^/day (± 2SE)		
Biscayne Bay	1	Fall 2017	148 (21)	164 (21)		–16 (16)	1.2 (0.2)	1.4 (0.2)	–0.1 (0.1)
	2	Summer 2018	307 (48)	305 (42)		2 (28)	2.5 (0.4)	2.5 (0.4)	0.0 (0.2)
	3	Spring 2019	174 (21)	164 (18)		9 (10)	1.4 (0.2)	1.4 (0.1)	0.1 (0.1)
	4	Fall 2019	187 (34)	204 (33)		–17 (18)	1.5 (0.3)	1.7 (0.3)	–0.1 (0.1)
	5^1^	Spring 2021	135 (32)	75 (23)		61 (15)	1.1 (0.3)	0.6 (0.2)	0.5 (0.1)
	All		189 (16)	185 (16)		4 (9)	1.6 (0.1)	1.5 (0.1)	0.0 (0.1)
Roman et al. (1983): NPP Results for 1977			9				0.07		
Seidensticker et al. (2019): NEP Results for 1997–2007						–15			–0.12
Tampa Bay	1	Fall 2017	236 (65)	255 (54)		–19 (41)	2.0 (0.5)	2.1 (0.4)	–0.2 (0.3)
	2	Summer 2018	217 (47)	262 (52)		–46 (27)	1.8 (0.4)	2.2 (0.4)	–0.4 (0.2)
	3^2^	Spring 2019	349 (152)	283 (114)		65 (76)	2.9 (1.3)	2.3 (0.9)	0.5 (0.6)
	4	Fall 2019	109 (25)	110 (22)		–1 (14)	0.9 (0.2)	0.9 (0.2)	0.0 (0.1)
	5	Spring 2021	164 (52)	181 (54)		–18 (19)	1.4 (0.4)	1.5 (0.4)	–0.1 (0.2)
	All		187 (27)	199 (26)		–12 (13)	1.5 (0.2)	1.6 (0.2)	–0.1 (0.1)
Johansson (2010): NPP Results for 1999–2008			136				1.12 in Hillsborough Bay		
			116				0.96 in Middle Tampa Bay		
			129				1.07 in Old Tampa Bay		

^1^Averages for deployment 5 in Biscayne Bay includes 3 out of 4 stations

^2^Averages for deployment 3 in Tampa Bay includes 3 out of 4 stations, all with observations lasting less than 5 days

Average GPP for all stations and deployments for Biscayne Bay and Tampa Bay are remarkably similar, 189 ± 16 and 187 ± 27 mmol O_2_ m^–2^ d^–1^, respectively (Table 3). Average ER for all stations and deployments for Biscayne Bay and Tampa Bay are also similar, 185 ± 16 and 199 ± 26 mmol O_2_ m^–2^ d^–1^, respectively. Overall, mean NEP for Biscayne Bay and Tampa Bay is neutral, within the estimated error, at 4 ± 9 and –12 ± 13 mmol O_2_ m^–2^ d^–1^, respectively (Table 3). In terms of carbon, average annual GPP for all stations and deployments is 570 ± 49 g C m^–2^ y^–1^ for Biscayne Bay and and 565 ± 81 g C m^–2^ y^–1^ for Tampa Bay.

## Discussion

### Comparisons with Previous Metabolism Studies

The scarcity of previous estuary metabolism studies for Biscayne Bay and Tampa Bay makes local comparisons of our findings challenging. In addition, two of the three previous studies estimated phytoplankton NPP (e.g., Johansson, 2010; Roman et al., 1983), which will always be less than GPP for the whole ecosystem (which is what we have estimated) because planktonic NPP is both limited to plankton (so does not include seagrass) and is a net rate.

Our average GPP estimate of 189 ± 16 mmol O_2_ m^–2^ d^–1^ in Biscayne Bay is much higher than planktonic NPP measured in 1977 (Roman et al., 1983), with a GPP:NPP ratio of 21. Historically, submerged vegetation has been shown to dominate primary production in Biscayne Bay (Roman et al., 1983), indicating that our much larger GPP estimate in this bay is likely due to contributions from seagrass. In the phytoplankton-dominated system of Tampa Bay, our mean GPP estimate of 187 ± 27 mmol O_2_ m^–2^ d^–1^ is around 60 mmol O_2_ m^–2^ d^–1^ higher than mean NPP from 1999–2008 determined by in-situ phytoplankton incubations using the ^14^C method (Johansson, 2010), resulting in a GPP:NPP ratio of ~ 1.5. The large difference between Biscayne Bay and Tampa Bay in their GPP:NPP ratios is likely a result of seagrass contributions to GPP in Biscayne Bay.

Seidensticker et al. (2019) noted that Biscayne Bay was typically net heterotrophic from 1997 to 2007 (mean NEP of –15 mmol O_2_ m^–2^ d^–1^), with a noteable exception after the 2005 hurricane season when the bay was temporarily net autotrophic. Compared with the long-term findings of Seidensticker et al. (2019), average NEP for our study is higher and not significantly different from zero (metabolically neutral) (Table 3, Fig. 5). This difference may be due to high interannual variability in NEP and low spatiotemporal coverage of our study relative to the long-term, system-wide approach of Seidensticker et al. (2019). Annual phytoplankton primary production, which is derived from both NPP and GPP estimates, was also previously observed to vary up to tenfold within an estuary and up to fivefold from year to year (Cloern et al., 2014). We are unaware of any other NEP studies in Tampa Bay.

### Environmental Drivers of Primary Production

Previous studies have observed several environmental factors that correlate to production and net metabolism in these and other estuaries. For example, nutrients and organic carbon were previously observed to be highest in Biscayne Bay during the wet season, defined as June–October, with higher dissolved oxygen % saturation in the dry season (Caccia & Boyer, 2005). In our study, however, the highest TN, TP, and chl-*a* concentrations were observed during the fall deployment in November 2017 (Table 2). Correlations between river discharge or precipitation and nutrients or chl-*a* concentrations in Biscayne Bay are difficult to establish because of high spatial variability and a lesser understood contribution from groundwater through the complex karst-dominated subsurface (Alarcon et al., 2022; Stabenau et al., 2015). Unlike the findings of Seidensticker et al. (2019) for NEP, average deployment GPP and nutrients per station (n = 19) had weak linear correlations in this study (Pearson correlation, *r*, between –0.13 and 0.16, p-values > 0.5).

Phytoplankton in Biscayne Bay have previously been identified as phosphorus-limited, whereas in Tampa Bay they are nitrogen-limited (Dixon et al., 2009; Johansson, 2010; Millette et al., 2019). In general, TN and TP concentrations in Tampa Bay and Biscayne Bay are within the range of recent published results (Corcoran et al., 2017; Greening et al., 2014; Millette et al., 2019). Also, our TN:TP ratios are consistent with prior notions of nutrient limitation, with TN:TP much greater than 16 in Biscayne Bay and TN:TP much less than 16 in Tampa Bay. In Biscayne Bay, our TN:TP ratios are also similar to the mean dissolved inorganic N:P ratio of around 100 (Millette et al., 2019). The low concentrations of TP in Biscayne Bay also resulted in water column C:P ratios up to around 1300, within range of previously observed C:P ratios up to about 1100 in seagrass biomass across the bay (Bourque & Fourqurean, 2013).

As previously noted, the final deployment period in Tampa Bay was approximately two weeks after a large influx of inorganic nitrogen to Lower Tampa Bay from the Piney Point facility (Beck et al., 2022). Metabolic rates for Tampa Bay during this period were similar to the previous deployments periods, suggesting a minimal influence of the nutrient influx on systemwide metabolism. A large diatom bloom of approximately ~ 25 km^2^ was observed in April in the vicinity of the wastewater release at Port Manatee on the southeast shore of Lower Tampa Bay. Stations TB4 and TB7 in Lower Tampa Bay were outside of the area of the initial bloom, perhaps explaining why metabolic rates during this period were similar to others. Unpublished metabolic estimates using in situ data from the weather station in Lower Tampa Bay (Fig. 2a) were similar to those herein (M.W. Beck, unpublished data, https://shiny.tbep.org/piney-point/lobo.Rmd). However, high bloom concentrations of the red tide organism *Karenia brevis* were observed in Middle and Lower Tampa Bay from June to July in 2021 after the last deployment of this study. Additional unpublished results from the weather station during this time showed production rates nearly three times higher than those herein.

Previous estuary metabolism studies along the Gulf of Mexico found that temperature, irradiance, chl-*a*, and/or salinity were strong predictors of GPP and/or NEP (Russell & Montagna, 2007; Caffrey et al., 2014; Murrell et al., 2018). In our study, the strongest linear correlation in Biscayne Bay is between temperature and GPP (*r* = 0.60, *p* < 0.001), whereas salinity has the strongest positive correlation with GPP and NEP in Tampa Bay (*r* = 0.39, *p* = 0.099 and 0.104, respectively) (Table 4). The salinity correlations with GPP and NEP in Tampa Bay are consistent with the salinity gradient in the bay, where salinity and metabolism tended to be lower in Old Tampa Bay (TB2) and higher in Lower Tampa Bay (TB4 and TB7). The temporal ranges of the aforementioned previous studies are greater than those of the study presented here, suggesting that the limited spatiotemporal resolution of this study makes it difficult to capture ecological responses to the observed variability in these environmental factors. Future studies should include (1) in-situ photosynthetically active radiation or total suspended solids measurements to elucidate the GPP and NEP relationships to light at this spatiotemporal scale and (2) a longer deployment periods to make statistics more robust.

**Table 4:** Pearson’s Correlations (ranging from –1 to 1) and *p-values* for means of GPP and mean NEP per station per deployment and the means of chlorophyll-a, water temperature, salinity, total phosphorus, total nitrogen, and total organic carbon per station per deployment for both Biscayne Bay and Tampa Bay. Correlations with *p-values* less than 0.100 are in bold

	Biscayne Bay		Tampa Bay	
	GPP	NEP	GPP	NEP
Chlorophyll-a	0.030 *0.912*	–0.334 *0.206*	0.171 *0.483*	**–0.554** *0.014*
Water Temperature	**0.602** < *0.001*	**0.169** *0.007*	0.324 *0.176*	–0.324 *0.176*
Salinity	0.166 *0.497*	–0.323 *0.178*	0.390 *0.099*	0.384 *0.104*
Total Phosphorus	–0.126 *0.642*	–0.051 *0.851*	0.067 *0.785*	**–0.478** *0.038*
Total Nitrogen	–0.046 *0.866*	–0.144 *0.594*	–0.084 *0.732*	–0.297 *0.217*
Total Organic Carbon	0.093 *0.731*	–0.126 *0.643*	–0.217 *0.437*	**–0.600** *0.018*

### Seagrass vs. Phytoplankton Dominance

Seagrass extent in Tampa Bay decreased from around 160 km^2^, or about 15% of the total area, in 1950 to about 90 km^2^ in 1982, mainly due to high nutrient loading into the bay that increased phytoplankton production and turbidity, eventually reducing light availability (Yarbro & Carlson 2011). A similar seagrass reduction has occurred more recently in Biscayne Bay. Hillsborough Bay, just north of station TB3, has a well-known history of poor water quality and a seagrass community with an historic extent of about 9 km^2^ that was nearly absent by the early 1980 s (Johansson & Greening, 1999; Yarbro and Carlson 2011). From 1988 to 2016, seagrass expanded by about 80 km^2^ across Tampa Bay due to improvements in water quality (Sherwood et al., 2017). Most of the seagrass recovery across the bay has occurred along the fringes of Middle Tampa Bay and Old Tampa Bay (Sherwood et al., 2017).

Primary productivity in Tampa Bay has historically been dominated by phytoplankton (Johansson, 2010; Johansson et al., 1985). Although seagrass is another organic matter producer in Tampa Bay, Johansson (2010) estimated that phytoplankton were responsible for roughly 95% of production in the bay in the 2000s. Phytoplankton production rates across Tampa Bay decreased from about 700 to around 400 g C m^–2^ y^–1^ from the early 1980 s to the late 2000 s due to improvements in water quality (Johansson, 2010), which is lower than our current Tampa Bay GPP estimate of 565 g C m^–2^ y^–1^.

**Fig. 7:**
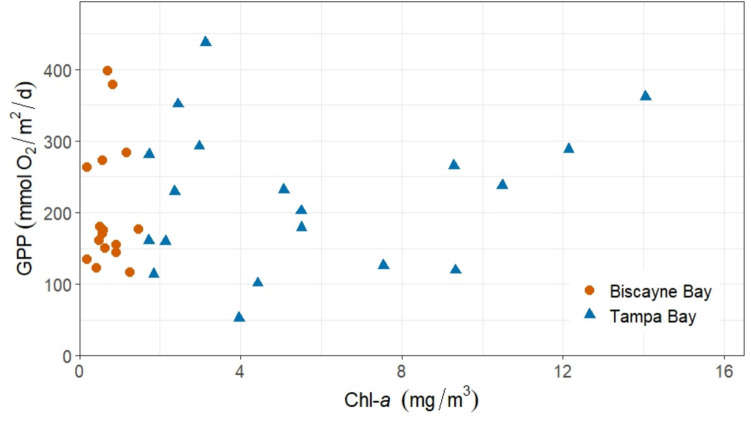
Mean gross primary production (GPP) for each station per deployment compared to mean chl-*a* for Tampa Bay and Biscayne Bay

Our measurements of chl*-a* concentrations are similar to those observed in the late 2000 s across Tampa Bay by Johansson (2010) and are negatively correlated with NEP (*r* = –0.55, *p* = 0.014) (Table 4), but the range of GPP associated with lower chl-*a* concentrations observed in this system supports that seagrass may be contributing more than previously observed in terms of productivity (Fig. 7). Some of the highest mean GPP estimated in this study occurred in Lower and Middle Tampa Bay when chl-*a* concentrations were below 5 mg m^–3^, suggesting higher contributions from seagrass at these stations. As water quality improves across Tampa Bay, eutrophication-driven algal blooms will decrease in frequency and extent, allowing seagrasses to continue expanding due to increased light availability.

Until the mid-2000s Biscayne Bay seagrass covered almost 650 km^2^, over 90% of the total bay area (Yarbro & Carlson 2011). Increases in TP and nitrate inputs into this oligotrophic system resulted in large algal blooms and subsequent seagrass die-off due to light limitation (Miami-Dade County, 2019). As of 2019, sub-basins in the northern end of Biscayne Bay, near stations BB1 and BB2, have lost up to 90% of seagrasses, whereas Manatee Bay to the south, near station BB4, has lost over 93% compared to coverage observed in 2005 (Miami-Dade County, 2019). Our finding of a weak correlation between GPP and chl-*a* in Biscayne Bay (Table 4; Fig. 7) suggests that GPP is still dominated by seagrass, not phytoplankton, regardless of the decrease in seagrass extent. Mean water temperature is strongly correlated to GPP in this system (*r* = 0.60, p < 0.001), with peak GPP occurring during the summer 2018 deployment, supporting that seagrass productivity is seasonally influenced.

### Comparisons to Regional and Global Estimates of Metabolism

Our mean GPP estimates of 570 ± 49 and 565 ± 81 g C m^–2^ y^–1^ for Biscayne Bay and Tampa Bay, respectively, tend to be higher than previous regional and global estimates (Table 5). For example, a study of the carbon budget of the United States east coast found that the estuarine NPP average in the South Atlantic Bight, including seagrass and plankton, is 386 ± 178 g C m^–2^ y^–1^ (Najjar et al., 2018).

**Table 5:** Comparison of results from this study to other regional and global metabolism estimates (± 2 standard errors if available)

Study	Location	Metabolism Estimates (± 2SE) g C m^–2^ y^–1^
Caffrey et al. (2014)	Gulf of Mexico, USA	GPP: 377–825
Cloern et al. (2014)	Global	APPP: 252
Murrell et al. (2018)	Pensacola Bay, Florida	GPP: 595
Najjar et al. (2018)	North America East Coast	NPP: 386 ± 178
This study	Biscayne Bay, Florida Tampa Bay, Florida	GPP: 570 ± 49 GPP: 565 ± 81

Caffrey (2004) utilized 5 years of water quality data collected from 42 stations within NERRs around the United States, including Puerto Rico, to examine what variables control ecosystem metabolism. Caffrey (2004) used a different conversion factor from oxygen to carbon compared to our study. As such, the Hedges et al. (2002) C:O_2_ ratio of 106:154 was applied to metabolism results from Caffrey (2004) for comparability to those herein. Out of the 42 stations, 39 were net heterotrophic with the highest NEP over –700 g C m^–2^ y^–1^ in Rookery Bay in the southern end of Florida (Caffrey, 2004). In a more recent study, Caffrey et al. (2014) used about 20 years’ worth of high-resolution water quality data from the NERRs, this time just along the northeastern Gulf of Mexico, to evaluate seasonal and interannual trends in historical metabolism. Average annual GPP was estimated to range between 377 and 825 g C m^–2^ y^–1^ with 27% of the productivity occurring in the benthos (Caffrey et al., 2014). Our GPP estimates of 570 and 565 g C m^–2^ y^–1^ are well within this range. Caffrey et al. (2014) also found that the spring and fall seasonal estimates of the average annual cycle of estuarine GPP were similar. We also did not observe clear seasonality in GPP between fall and spring deployments in either Biscayne or Tampa Bay (Table 3). Another estuary metabolism study, although on a much shorter timescale of less than a year, from Pensacola Bay, Florida, found a similar mean GPP of about 595 g C m^–2^ y^–1^, with also nearly balanced NEP, with significant differences between the spring and summer seasons at the deeper offshore site (Murrell et al., 2018). Comparing more modern studies in estuaries that have nearly balanced NEP to estuaries with such high estimates from up to twenty years ago illustrates the importance of monitoring estuarine metabolism that is continually undergoing changes in the face of anthropogenic pressures, such as climate change.

**Fig. 8:**
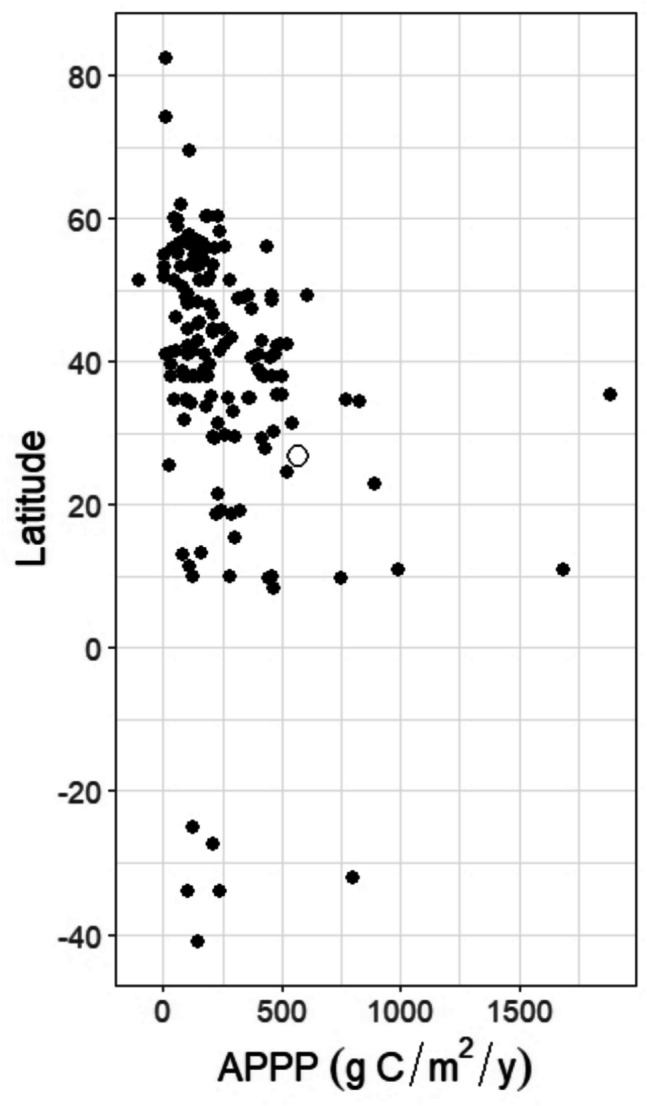
Comparison of our GPP estimates (open circle) with the global synthesis of annual phytoplankton primary productivity estimates from Cloern et al., (2014; closed circles)

The “global” mean annual planktonic primary production (APPP), which includes NPP, GPP, and undefined production estimates for estuarine–coastal ecosystems, but not the results from Caffrey (2004) or more recent publications, is 252 g C m^–2^ y^–1^ (Cloern et al., 2014). Compared to this global APPP study, GPP for Tampa Bay and Biscayne Bay is over twice the APPP average. Cloern et al. (2014) recognize the wide range of production values in their literature synthesis, which is driven primarily by studies from the Baltic and North America, skewing the mean and median (185 g C m^–2^ y^–1^) to values that may not be globally representative (Fig. 8).

## Conclusions

In this study we made the first systemwide estimates of gross primary production and respiration in Tampa Bay and Biscayne Bay, two large subtropical estuaries of the southeastern United States. Comparing our results to previous studies conducted in these estuaries via GPP:NPP ratios of GPP to planktonic NPP shows that Biscayne Bay GPP is about 20 times higher than planktonic NPP, supporting that primary production is dominated by seagrass. However, in Tampa Bay this ratio is only 1.5, suggesting phytoplankton have a much higher contribution to production in Tampa Bay than in Biscayne Bay. Despite differences in dominant primary producers, Biscayne Bay and Tampa Bay have similar mean GPP estimates of 570 ± 49 and 565 ± 81 g C m^–2^ y^–1^, respectively. Mean NEP is nearly balanced in both of these systems, but this is likely to change in the future with increasing nutrient loading in Biscayne Bay and shifts in seagrass extent in both bays. GPP estimates for both bays fall within the range of other Gulf of Mexico estuarine metabolism studies, but our production results tend to be on the higher end of estimates from other regions of the United States and on a global scale. Although previous studies observe summer peaks in the annual mean cycle of GPP, our study does not show strong seasonality, especially between spring and fall deployments. The “global” mean APPP is 252 g C m^–2^ y^–1^ (Cloern et al., 2014), but GPP for Tampa Bay and Biscayne Bay is over twice this average. This difference and the lack of estuarine metabolism studies in other global subtropical regions highlight the importance of more frequent production measurements in these complex estuaries, especially in the face of a changing climate. Water chemistry and discrete sample data are available online at 10.26207/zvsy-yd62 (Briceño et al. 2022).

## Supplementary Information

Below is the link to the electronic supplementary material.

Supplementary file1 (DOCX 22 KB)

Supplementary file2 (DOCX 32 KB)
