# Supplementary material for: Ecosystem Metabolic Rates Estimated from Diel Oxygen Measurements in Two Subtropical Estuaries
Source: Estuaries Coast. 2025 Aug 7;48(6):155. doi: 10.1007/s12237-025-01597-y (PMC12331821; doi:10.1007/s12237-025-01597-y)
Supplement: Supplementary file 1 — Supplementary file1 (DOCX 22 KB) [file 12237_2025_1597_MOESM1_ESM.docx]

Ecosystem Metabolic Rates Estimated from Diel Oxygen Measurements in Two Subtropical Estuaries

J.M. Arriola, R.G. Najjar*, H. Briceño, C. Hu, M. Herrmann, and M.W. Beck

*Pennsylvania State University; rgn1@psu.edu

**Table S1.** Mean water depths (m) ± 2 standard error for each station for all deployments and means for all stations and deployments per bay.

| **Estuary** | **Station** | **Mean Water Depth (m)**  (±2SE) |
| --- | --- | --- |
| Biscayne Bay | BB1 | 3.81 (0.01) |
|  | BB2 | 3.60 (0.01) |
|  | BB3 | 3.43 (0.01) |
|  | BB4 | 3.10 (0.01) |
|  | Mean | 3.47 (0.00) |
| Tampa Bay | TB1 | 3.53 (0.01) |
|  | TB2 | 1.54 (0.01) |
|  | TB3 | 2.95 (0.01) |
|  | TB4 | 3.38 (0.01) |
|  | TB7 | 1.95 (0.01) |
|  | Mean | 2.84 (0.01) |
