# Supplementary material for: Ecosystem Metabolic Rates Estimated from Diel Oxygen Measurements in Two Subtropical Estuaries
Source: Estuaries Coast. 2025 Aug 7;48(6):155. doi: 10.1007/s12237-025-01597-y (PMC12331821; doi:10.1007/s12237-025-01597-y)
Supplement: Supplementary file 2 — Supplementary file2 (DOCX 32 KB) [file 12237_2025_1597_MOESM2_ESM.docx]

Ecosystem Metabolic Rates Estimated from Diel Oxygen Measurements in Two Subtropical Estuaries

J.M. Arriola, R.G. Najjar*, H. Briceño, C. Hu, M. Herrmann, and M.W. Beck

*Pennsylvania State University; rgn1@psu.edu

**Table S2.** Minima, maxima, average, and ±2 standard errors of water temperature, salinity, and dissolved oxygen for all stations per deployment.

| Estuary | Deployment | Station ID | Temperature (°C) | | | | Salinity (psu) | | | | Dissolved Oxygen (mmol/m^3^) | | | |
| --- | --- | --- | --- | --- | --- | --- | --- | --- | --- | --- | --- | --- | --- | --- |
|  |  |  | Min | Max | Avg | 2SE | Min | Max | Avg | 2SE | Min | Max | Avg | 2SE |
| Biscayne Bay | **1**  Fall 2017 | BB1 | 25.1 | 26.9 | 26.0 | *0.02* | 29.9 | 33.4 | 31.5 | *0.03* | 177.8 | 233.4 | 209.4 | *0.6* |
|  |  | BB2 | 24.7 | 26.4 | 25.6 | *0.02* | 26.2 | 29.1 | 27.6 | *0.03* | 182.8 | 271.6 | 216.3 | *0.9* |
|  |  | BB3 | 24.9 | 26.9 | 26.1 | *0.02* | 26.5 | 29.7 | 28.4 | *0.04* | 182.2 | 254.1 | 216.9 | *0.6* |
|  |  | BB4 | 24.9 | 27.0 | 26.0 | *0.03* | 20.0 | 25.8 | 22.1 | *0.09* | 185.9 | 276.6 | 223.8 | *0.9* |
|  | **2**  Summer 2018 | BB1 | 30.4 | 32.1 | 31.2 | *0.02* | 32.0 | 33.3 | 32.5 | *0.02* | 175.3 | 267.5 | 208.4 | *0.9* |
|  |  | BB2 | 30.4 | 32.1 | 31.2 | *0.02* | 30.0 | 32.6 | 31.2 | *0.03* | 163.4 | 262.2 | 216.6 | *1.3* |
|  |  | BB3 | 30.6 | 32.4 | 31.3 | *0.03* | 34.3 | 35.9 | 35.1 | *0.02* | 148.4 | 255.6 | 197.5 | *1.3* |
|  |  | BB4 | 30.8 | 32.4 | 31.5 | *0.02* | 31.3 | 32.9 | 31.9 | *0.03* | 140.0 | 247.2 | 197.2 | *1.3* |
|  | **3**  Spring 2019 | BB1 | 24.2 | 28.1 | 26.1 | *0.04* | 35.8 | 38.0 | 37.0 | *0.02* | 179.4 | 249.1 | 207.5 | *0.6* |
|  |  | BB2 | 24.3 | 28.2 | 26.0 | *0.05* | 34.9 | 38.2 | 36.4 | *0.04* | 181.3 | 270.0 | 221.9 | *0.6* |
|  |  | BB3 | 24.6 | 27.2 | 26.0 | *0.03* | 39.0 | 39.4 | 39.2 | *0.00* | 196.9 | 247.5 | 214.7 | *0.3* |
|  |  | BB4 | 25.2 | 28.7 | 26.7 | *0.04* | 39.7 | 40.2 | 40.0 | *0.00* | 184.1 | 252.2 | 205.9 | *0.6* |
|  | **4**  Fall 2019 | BB1 | 27.8 | 29.6 | 28.7 | *0.02* | 30.6 | 34.3 | 33.2 | *0.03* | 159.7 | 242.2 | 200.9 | *0.6* |
|  |  | BB2 | 27.7 | 29.5 | 28.6 | *0.02* | 22.9 | 36.2 | 28.6 | *0.03* | 168.8 | 240.0 | 200.3 | *0.6* |
|  |  | BB3 | 25.1 | 29.6 | 27.8 | *0.06* | 31.2 | 36.3 | 35.2 | *0.03* | 49.1 | 246.3 | 195.0 | *0.6* |
|  |  | BB4 | 25.2 | 29.6 | 27.7 | *0.06* | 27.5 | 35.8 | 34.9 | *0.02* | 139.7 | 246.3 | 192.5 | *0.6* |
|  | **5**  Spring 2021 | BB1 | 27.5 | 29.8 | 28.7 | *0.03* | 25.0 | 25.7 | 25.3 | *0.01* | 208.1 | 270.9 | 239.7 | *0.6* |
|  |  | BB2 | 27.5 | 29.9 | 28.7 | *0.03* | 22.8 | 24.5 | 24.2 | *0.01* | 215.0 | 291.3 | 251.3 | *1.3* |
|  |  | BB3 | 27.8 | 29.6 | 28.8 | *0.02* | *NA* | *NA* | *NA* | *NA* | 194.4 | 262.8 | 238.1 | *0.6* |
|  |  | BB4 | 27.7 | 29.8 | 28.7 | *0.03* | 23.6 | 24.5 | 24.0 | *0.01* | 173.4 | 246.3 | 231.3 | *0.6* |
| Tampa Bay | **1**  Fall 2017 | TB1 | 27.4 | 28.6 | 28.0 | *0.02* | 20.5 | 23.6 | 21.9 | *0.05* | 150.3 | 294.4 | 218.8 | *1.9* |
|  |  | TB2 | 25.9 | 30.6 | 28.0 | *0.04* | 14.7 | 20.6 | 18.8 | *0.08* | 181.3 | 300.9 | 215.6 | *1.3* |
|  |  | TB3 | 27.5 | 28.8 | 28.0 | *0.00* | 22.8 | 23.5 | 23.2 | *0.00* | 150.0 | 265.6 | 205.6 | *0.3* |
|  |  | TB4 | 27.5 | 28.8 | 28.0 | *0.02* | 28.3 | 31.3 | 29.7 | *0.06* | 172.2 | 227.8 | 196.3 | *0.9* |
|  | **2**  Summer 2018 | TB1 | 29.5 | 31.3 | 30.4 | *0.03* | 20.5 | 26.6 | 23.8 | *0.12* | 145.0 | 230.9 | 187.2 | *0.9* |
|  |  | TB2 | 29.1 | 32.1 | 30.6 | *0.04* | 21.9 | 25.0 | 23.2 | *0.05* | 145.6 | 271.9 | 189.4 | *1.3* |
|  |  | TB3 | 29.5 | 31.8 | 30.7 | *0.03* | 23.1 | 26.0 | 23.9 | *0.04* | 44.1 | 240.9 | 167.8 | *1.6* |
|  |  | TB4 | 29.5 | 31.4 | 30.3 | *0.03* | 28.6 | 32.5 | 31.2 | *0.04* | 158.1 | 218.4 | 187.8 | *0.9* |
|  | **3**  Spring 2019 | TB1 | 29.1 | 29.9 | 29.5 | *0.02* | 28.2 | 29.5 | 28.6 | *0.04* | 185.0 | 225.9 | 207.5 | *0.9* |
|  |  | TB2 | 28.4 | 30.5 | 29.5 | *0.03* | 24.4 | 27.0 | 25.6 | *0.07* | 171.9 | 282.5 | 216.6 | *2.5* |
|  |  | TB7 | 28.2 | 30.9 | 29.2 | *0.04* | 30.8 | 34.9 | 33.4 | *0.09* | 105.6 | 337.5 | 195.0 | *2.8* |
|  | **4**  Fall 2019 | TB1 | 19.9 | 21.1 | 20.6 | *0.05* | 24.7 | 26.9 | 25.8 | *0.10* | 214.4 | 273.8 | 253.1 | *1.6* |
|  |  | TB2 | 18.3 | 21.8 | 20.2 | *0.03* | 20.0 | 23.9 | 21.8 | *0.04* | 205.9 | 283.8 | 240.9 | *0.6* |
|  |  | TB3 | 18.3 | 22.0 | 20.7 | *0.03* | 22.0 | 26.5 | 25.7 | *0.01* | 207.8 | 275.0 | 232.2 | *0.6* |
|  |  | TB7 | 19.0 | 22.3 | 20.8 | *0.03* | 24.2 | 30.5 | 28.4 | *0.04* | 196.6 | 346.9 | 255.3 | *0.9* |
|  | **5**  Spring 2021 | TB1 | 24.9 | 28.9 | 26.7 | *0.06* | 20.4 | 23.9 | 21.5 | *0.04* | 139.4 | 301.3 | 230.9 | *1.3* |
|  |  | TB2 | 24.9 | 29.6 | 27.1 | *0.06* | 17.9 | 20.3 | 18.8 | *0.04* | 100.3 | 267.5 | 212.5 | *1.9* |
|  |  | TB3 | 24.8 | 28.8 | 26.8 | *0.06* | 17.7 | 22.4 | 20.5 | *0.05* | 156.3 | 274.4 | 235.3 | *1.3* |
|  |  | TB7 | 24.8 | 30.5 | 27.1 | *0.07* | 18.8 | 22.1 | 20.4 | *0.05* | 122.8 | 381.3 | 249.7 | *2.5* |
